# Aquaporin 1 and 3 as local vitality markers in mechanical and thermal skin injuries

**DOI:** 10.1007/s00414-021-02588-x

**Published:** 2021-04-14

**Authors:** Julian Prangenberg, E. Doberentz, A. -L. Witte, B. Madea

**Affiliations:** Institute of Legal Medicine, University Hospital Bonn, Stiftsplatz 12, 53111 Bonn, Germany

**Keywords:** Aquaporin 1, Aquaporin 3, Immunohistochemistry, Wound vitality, Forensics

## Abstract

Assessment of the vitality of an injury is one of to the main tasks in daily forensic casework. Aquaporins belong to the family of water channels. They enable the transport of water and of small molecules like glycerol through biological channels. So far, 13 classes of aquaporins are identified in vertebrates. The classical aquaporin channels 1, 2 and 4 are only permeable for water. The aquaporin channels 3, 7, 9 and 10 are also called aquaglycerolporins since they can also transport glycerol. Aquaporin 3 is expressed in epidermal keratinocytes. In the present investigation, the aquaporin 1 and 3 expression in mechanically and thermally damaged skin is investigated by immunohistochemistry. The study collective comprises 30 cases (63.3% male and 36.7% female) with an age range between 19 and 95 years (mean value 54.6 years). The skin injury comprises different kinds of blunt force, sharp force, strangulation marks, thermal injury, gunshot wounds and frost erythema. In all kinds of mechanical and trauma injury, an increased expression of aquaporin 3 in the keratinocytes of the epidermis was found. There is no correlation of the aquaporin 3 expression with age, sex, body mass index, duration of agonal period and postmortem interval. Concerning aquaporin 1, there were no differences between injured and uninjured skin. Aquaporin 3 is independently from the kind of skin injury and appears to be a valuable immunohistochemical parameter of vitality.

## Introduction

Assessment of the vitality of an injury belongs to the main tasks in daily forensic casework. Forensic specialists are often confronted with the questions whether injuries occurred antemortem or postmortem and how long a person might have survived those [1]. Furthermore, inflicted injuries may not be clearly visible. For example, strangulation marks may only be vaguely formed and even vanish over time [2, 3].

In the forensic context, wound vitality is closely related to tissue repair. In this context, the early tissue repair that occurs right after an injury may also be described as vital reaction [1]. The process of wound healing is quite similar in different tissues and may be subdivided into different phases. Due to this similar process, a determination of the wound-age is possible. The wound-age corresponds with the time between the trauma and time of death. Eventually, it describes the time of survival of an individual [4].

The estimation of wound vitality may be accomplished with immunohistochemical stainings and/or determination of gene expression of proteins like aquaporins [5].

Aquaporins belong to the family of water channels. They enable the transport of water and of small molecules like glycerol through biological channels. So far, 13 classes of aquaporins are identified in vertebrates. The classical aquaporins (AQP 1, 2, 4) are only permeable for water. The aquaporin channels 3, 7, 9 and 10 are also called aquaglycerolporins since they can also transport glycerol [6]. AQP1 is located around the dermal capillaries [7]. Aquaporin 3 is expressed in epidermal keratinocytes [8]; the stratum corneum of the epidermis does not contain keratinocytes and AQP3-channels [9].

AQP3 is also involved in the process of wound healing. A study on AQP3-deficient mice showed that those mice had less epidermal cell migration to the wound area compared to wild-type mice.

Ishida et al. [10] investigated the aquaporin expression in 56 strangulation cases. They found a positive expression in the strangulation mark for aquaporin 3. Additionally, AQP1 and AQP3 can be used to further determine the wound age [11]. AQP3 also proved to be a reliable forensic marker in burning victims. It has been shown, that AQP3 is missing in the center of a burning wound and that there is an enhanced expression of AQP at the margin of the wound [12].

In the present investigation, the aquaporin 1 and 3 expression in mechanically and thermally damaged skin is investigated by immunohistochemistry.

## Material and methods

The study collective comprises 30 cases (63.3% male and 36.7% female) with an age range between 19 and 95 years (mean value 54.6 years). A detailed overview is shown in Table 1. The skin injury comprises different kinds of blunt force, sharp force, strangulation marks, thermal injury, frost erythema and gunshot wounds. The duration of agony was defined as very short (less than a minute), short (less than an hour) and long (more than an hour). In each case, a sample of the macroscopically uninjured abdominal skin was taken to compare the expression of AQP. Cases of polytrauma and decay were excluded from this study. An immunohistochemical staining was performed by using the following antibodies: mouse anti-AQP1 monoclonal antibody and rabbit anti-AQP3 polyclonal antibody (Abcam, Cambridge, United Kingdom). In accordance to manufacturer’s instructions, visualization of the immune complexes was done by using Dako Envision + Dual Link System (Dako Denmark, Glostrup, Denmark).

**Table 1:** Detailed overview of the AQP3 study collective

No	Injury	Cause of death	sex	age	weight (kg)	height (cm)	Body mass index	Duration of agony	Longest possible postmortem interval until autopsy (days)	Average staining of uninjured skin (%)	Average staining of injured skin (%)	Resuscitation
1	Excoriation	Traumatic brain injury	M	49	49	165	17.9	Short	5	20	100	Yes
2	Dried skin abrasions	Suffocation	F	89	74	162	28.2	Short	2	50	80	No
3	Dried skin abrasions	Pulmonary embolism	F	67	47	157	18.9	Long	1	50	60	Yes
4	Dried skin abrasions	Internal bleeding	M	58	107	185	31.2	Long	3	65	100	Yes
5	Dried skin abrasions	Amniotic fluid embolism	F	29	83	160	32.4	Long	4	75	100	Yes
6	Dried skin abrasions	Endocarditis caused by drug abuse	M	36	84	175	27.4	Long	3	52	72	Yes
7	Dried skin abrasions	Repeat heart attack	M	73	130	183	38.8	Long	5	50	85	Yes
8	Dried skin abrasions	Repeat heart attack	M	74	60	170	20.9	Long	5	80	40	Yes
9	Dried skin abrasions	Thoracic trauma	M	19	76	181	23.2	Long	4	20	70	No
10	Frost erythema	Hypothermia	M	58	62	172	21.1	Long	19	51	80	No
11	Frost erythema	Hypothermia	F	68	80	164	29.7	Long	9	62	93	No
12	Laceration	External bleeding	M	45	65	180	20.1	Long	5	0	50	No
13	Laceration	External bleeding	F	73	78	174	25.7	Short	2	51	77	Yes
14	Laceration	External bleeding	M	42	87	176	28.1	Short	9	15	70	No
15	Laceration	Traumatic brain injury	M	55	104	175	33.8	Short	3	53	100	Yes
16	Laceration	Heart attack	M	66	84	175	27.4	Short	7	70	40	No
17	Laceration	Suspected cardiac arrest	m	58	81	172	27.4	Long	9	100	100	No
18	Stab wound	Internal bleeding	F	39	43	150	19.1	Short	3	60	48	No
19	Stab wound	Internal bleeding	m	58	70	166	25.4	Short	5	100	100	No
20	Stab wound	Drowning	m	59	75	163	28.2	Short	4	0	40	No
21	Stab wound	Internal bleeding	w	53	60	177	19.2	Short	2	100	100	Yes
22	Gunshot wound	External bleeding	M	40	71	180	21.8	Short	< 1	15	90	No
23	Gunshot wound	Penetrating brain injury	M	95	68	173	22.7	Very short	8	35	15	No
24	Strangulation mark	Suffocation	M	35	105	170	36.4	Short	3	14	100	No
25	Strangulation mark	Hanging	M	42	73	175	23.8	Short	6	15	10	Yes
26	Strangulation mark	Hanging	m	55	85	178	26.8	Short	3	65	80	No
27	Strangulation mark	Hanging	m	48	61	167	21.9	Short	5	65	100	No
28	Strangulation mark	Hanging	m	43	67	161	25.8	Short	2	100	100	Yes
29	Strangulation mark	Hanging	m	72	75	170	26.0	Short	4	80	80	No
30	Strangulation mark	Hanging	w	39	45	162	17.1	Short	7	100	100	No

**Fig. 1:**
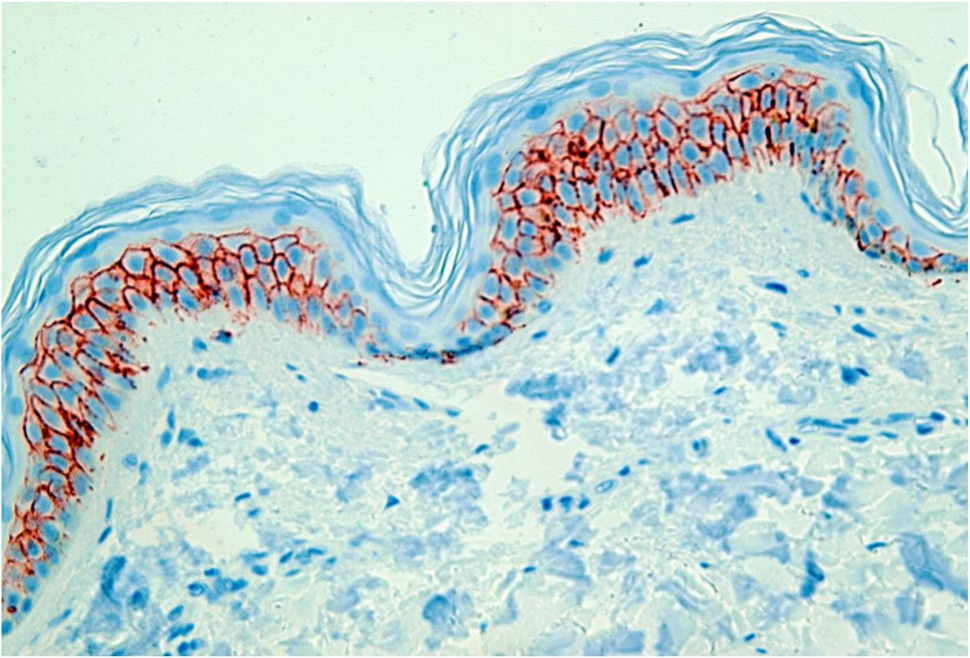
Study collective AQP3, bullet wound, 100% expression, magnification of 400X

**Fig. 2:**
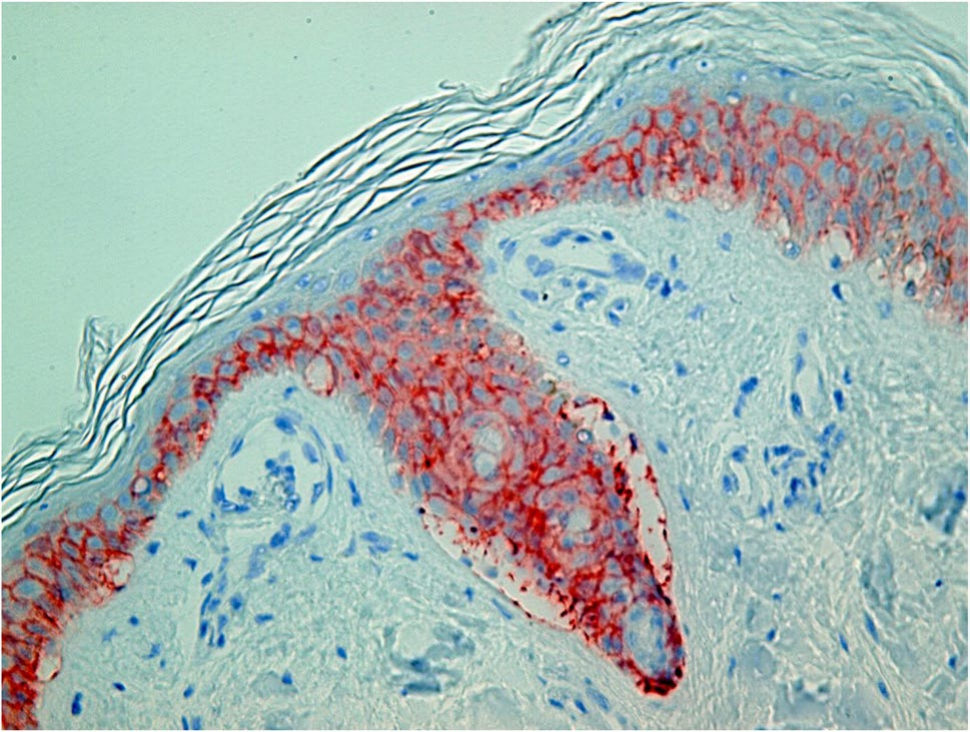
Study collective AQP3, frost erythema, 100% expression, magnification of 400X

**Fig. 3:**
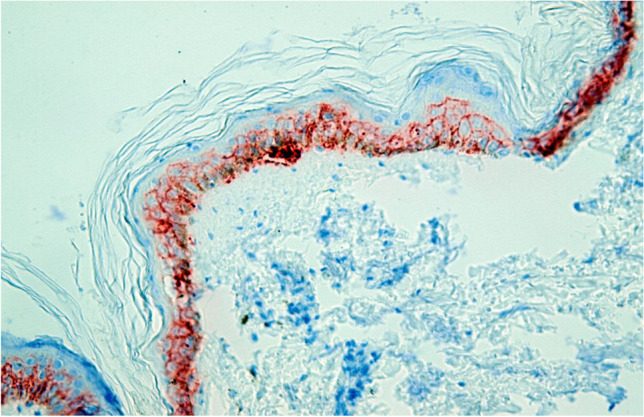
Study collective AQP3, laceration, 100% expression, magnification of 400X

**Fig. 4:**
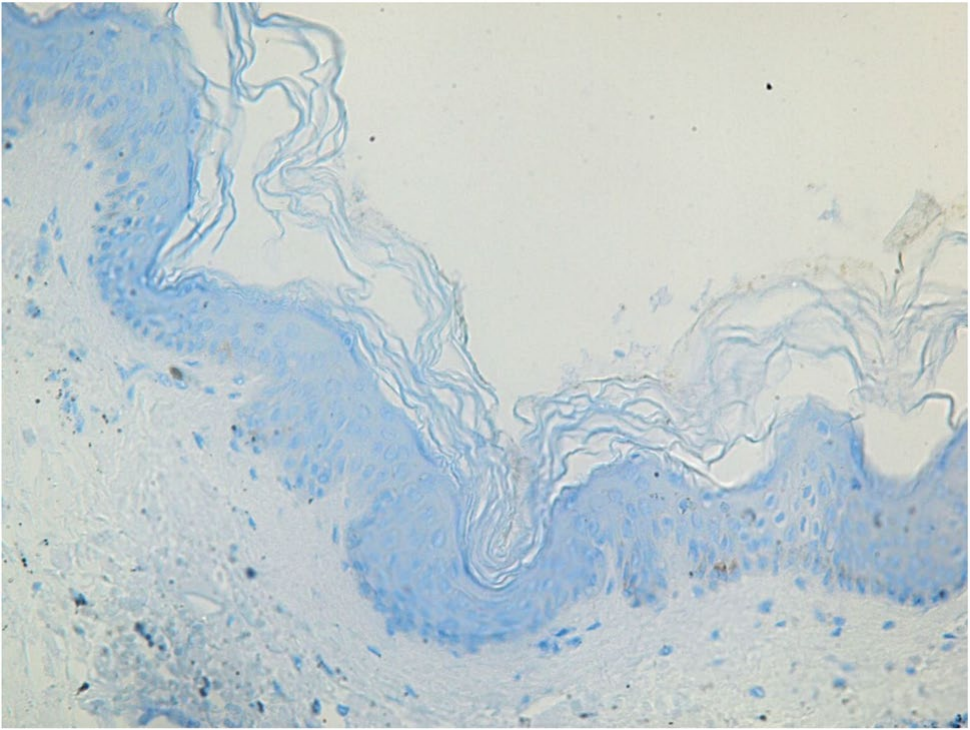
Control collective AQP3, no visible expression of the macroscopically uninjured abdominal skin, magnification of 400X

**Fig. 5:**
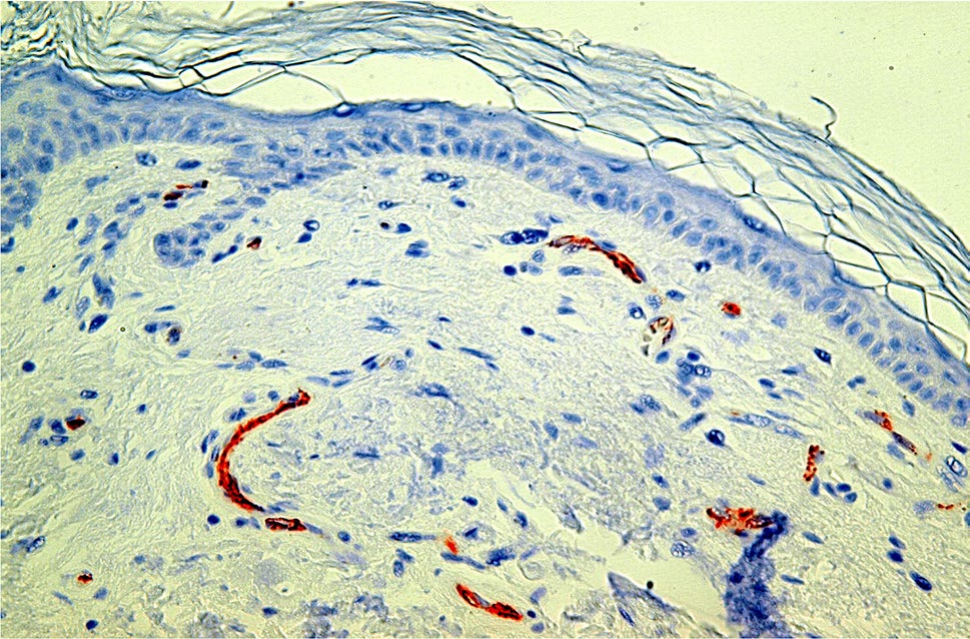
Study collective AQP1, notable expression on dermal capillaries but no visible expression in the epidermis of desiccated skin due to reanimation, magnification of 400X

After staining and preparation, slides were microscopically and semi-quantitatively analyzed wherein 10 randomly chosen high power fields (HPF) were observed by two independent investigators under a light microscope at 400 × magnification to assess reddish-stained AQP in the epidermal tissue (Figs. 1, 2, 3, 4 and 5). For each HPF, the ratio between the reddish-stained AQP in the epidermis and the unstained epidermis was estimated in percent and the mean value for all such HPFs was calculated. A 95% confidence interval was chosen and a Mann–Whitney-U-test for two independent samples was carried out. We also tested the strength of association between the staining in injured and uninjured skin and the kind of injury, sex and duration of agony by using the Eta Coefficient test and the correlation between the staining and age, body mass index and postmortem interval by using the Pearson correlation coefficient.

## Results

**Fig. 6:**
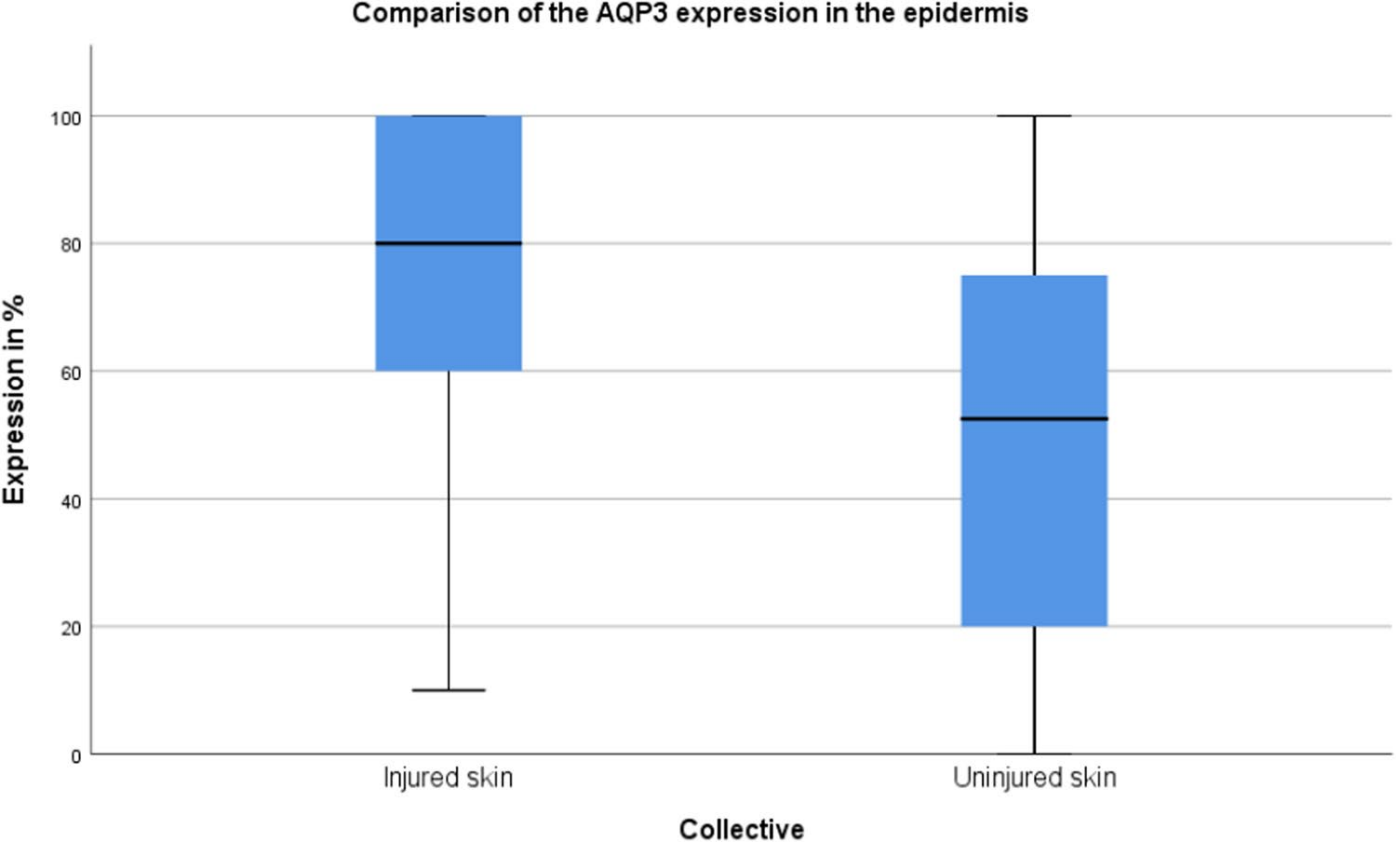
Comparison of the AQP3 expression in the epidermis in injured and uninjured skin

In all kinds of mechanical injuries and exposure to cold, an increased expression of aquaporin 3 in the keratinocytes of the epidermis was found. In the injured skin, a mean expression of 76.0% (min. 10%, max. 100%, median 80%, standard deviation 26.7) was observed compared to 53.8% (min. 0%, max. 100%, median 52.5%, standard deviation 30.9) in the uninjured skin (Fig. 6). Based on the result of the Mann–Whitney-U-test (p = 0,007), the AQP3 expression was significantly higher in injured skin compared to the uninjured skin.

There was no significant correlation between the intensity of AQP3 expression and age, sex, BMI, duration of agonal period or postmortem interval in injured and uninjured skin. The intensity of AQP3 expression was independent from the kind of injury. Concerning AQP1, there was no expression in the epidermis of injured and uninjured skin.

Resuscitation was performed in 12 of the 30 cases. In those cases, there was only a slight difference regarding the mean expression in injured as well as uninjured skin. The mean expression in the epidermis was 59.3% in uninjured and 78.7% in injured skin in cases where resuscitation was performed compared to 50.1% and 74.2%, respectively.

## Discussion

APQ3 showed an increased expression in the epidermis in all kinds of mechanical injuries and exposure to cold. In most cases, the expression was higher than in macroscopically uninjured skin. The expression in uninjured skin was higher than in the injured skin in only four cases and the injured skin never showed a negative expression. Regarding those four cases, cardiopulmonary resuscitation was performed or, as in one case, the person was found in abdominal position.

It also has to be considered, that there might has been some kind of blunt force impact on the abdominal area prior to the death of those people that caused an increased expression. This might be considered in case 18 were the woman died in a homicide due to multiple stab wounds. It also showed, that in cases of polytrauma, there was an overall increased and indistinguishable expression in injured and macroscopically uninjured skin.

Therefore, in cases of high impact forces, an immunohistochemical examination of the AQP3 expression does not seem useful. In cases of resuscitation, it has to be considered that there may be an overall increased AQP3 expression even in the uninjured skin. In our investigated cases, information was often missing whether the resuscitation was predominantly performed manually or mechanically. Further studies are needed to investigate a potential influence of the resuscitation technique on the overall AQP3 expression. In cases of short survival time (agonal period less than several minutes), it has to be considered that a low immunohistochemical AQP3 expression may also result from an incomplete AQP3 translation [5]. For those reasons, it still seems advisable to combine immunohistochemical investigations and determination of AQP gene expression to assess wound vitality.

Regarding the study by Ishida et al. [10], there was also a significant correlation between the AQP3 expressions in injured skin compared to uninjured skin. In contrast to their study, we found a higher mean AQP3 expression in the uninjured skin. This might be due to the fact, that we observed a smaller and very heterogeneous case collective and not only strangulation cases. Similar to the findings of Ishida et al., there seems to be no remarkable influence of postmortem changes regarding immunoreactivity of AQP3.

In conclusion, AQP3 expression is independent from the kind of skin injury and appears to be a valuable immunohistochemical parameter of wound vitality whereas AQP1 appears to be an unsuitable marker. Yet, there seem to be certain constellations where the use and significance of this examination may be restricted. Further studies are needed to establish a sufficient sensitivity and specificity of this method, to identify unsuitable constellations, to consolidate the validity in applicable scenarios and to find new suitable markers that in combination enhance the validity of immunohistochemical wound vitality determination. This could be achieved, for example, through a quantitative whole slide analysis of the skin samples since a semi-quantitative analysis like used in this study contains the risk of inaccuracies. Ultimately, there is still a need for comparison between those cases presented in this study and cases of confirmed postmortem injuries like animal scavenging or postmortem boat propeller injuries in order to rule out that postmortem changes occur in the AQP expression pattern.

## Data Availability

Not applicable.

## References

[1] Grellner W, Madea B (2007) Demands on scientific studies. Vitality of wounds and wound age estimation. Forensic Sci Int 165(2–3). 10.1016/j.forsciint.2006.05.029 10.1016/j.forsciint.2006.05.029 16806766

[2] Adelson L, Thomas C (1974). Homicide by cervical compression and by drowning “Asphyxial Deaths”. The Pathology of Homicide.

[3] Gordon I, Shaphiro HA, Berson SD (1988). Death usually initiated by hypoxic hypoxia or anoxic anoxia. Forensic medicine-a guide to principles.

[4] Kondo T, Ishida Y (2010). Molecular pathology of wound healing. Forensic Sci Int.

[5] Kubo H, Hayashi T, Ago K (2014). Forensic diagnosis of ante- and postmortem burn based on aquaporin-3 gene expression in the skin. Leg Med (Tokyo).

[6] Hara-Chikuma M, Verkman AS (2008). Aquaporin-3 facilitates epidermal cell migration and proliferation during wound healing. J Mol Med (Berl).

[7] Mobasheri A, Marples D (2004). Expression of the AQP-1 water channel in normal human tissues. A semiquantitative study using tissue microarray technology. Am J Physiol Cell Physiol.

[8] Sougrat R, Morand M, Gondran C (2002). Functional expression of AQP3 in human skin epidermis and reconstructed epidermis. J Invest Dermatol.

[9] Matsuzaki T, Suzuki T, Koyama H (1999). Water channel protein AQP3 is present in epithelia exposed to the environment of possible water loss. J HistochemCytochem.

[10] Ishida Y, Kuninaka Y, Nosaka M (2018). Forensic application of epidermal AQP3 expression to determination of wound vitality in human compressed neck skin. Int J Legal Med.

[11] Ishida Y, Kuninaka Y, Furukawa F (2018). Immunohistochemical analysis on aquaporin-1 and aquaporin-3 in skin wounds from the aspects of wound age determination. Int J Legal Med.

[12] Sebastian R, Chau E, Fillmore P (2015). Epidermal aquaporin-3 is increased in the cutaneous burn wound. Burns.

